# Impact of ultrasonography on central venous catheter insertion in intensive care

**DOI:** 10.4103/0971-3026.54877

**Published:** 2009-08

**Authors:** Gopal B Palepu, Juneja Deven, M Subrahmanyam, S Mohan

**Affiliations:** Department of Anesthesia and Critical Care Medicine, Global Hospital, Lakdi-ka-pul, Hyderabad, India

**Keywords:** Central venous catheters, internal jugular vein, subclavian vein, ultrasound-guided central lines

## Abstract

**Background and Aims:**

The insertion of central venous catheters (CVCs) is an integral part of the management of critically ill patients. We aimed to study the impact of ultrasonography (USG) on CVC insertion in intensive care.

**Setting and Design:**

A prospective study of 450 patients requiring CVC in the intensive care unit (ICU) of a tertiary care hospital.

**Methods and Materials:**

The patients were randomized into two groups: to have CVC insertion with USG-guidance or with the anatomic landmark technique (ALT). Data were collected on patient demographics; operator experience; and method, site and side of insertion. Outcome measures included successful insertion of CVC, number of attempts needed and complications.

**Results:**

Internal jugular vein (IJV) cannulation was successful in 177/194 patients (91.2%) using ALT and in 200/205 patients (97.6%) using USG guidance, a significant difference of 6.4% (*P* = 0.006). Using ALT, 72.7% of cannulations could be accomplished in the first attempt as compared with 84.4% with USG guidance (*P* = 0.004). The overall complication rate was 28/399 (7%), with 19 (9.8%) complications in the ALT group and 9 (4.4%) in the USG group (age-, sex-, and operator-adjusted OR = 0.35, 95% CI: 0.13–0.96; *P* = 0.03). For subclavian vein catheterization, the success rate was 26/28 (92.9%) in the ALT group and 17/17 (100%) in the USG group (*P* = 0.52). Using ALT, 71.4% cannulation could be accomplished in the first attempt as compared with 82.4% under USG guidance (*P* = 0.49). The overall complication rate was 6/45 (13.3%), with 4 (14.3%) complications in the ALT group and 2 (11.8%) in the USG group (*P* > 0.99).

**Conclusions:**

Real-time USG guidance improves success rates, reduces the number of attempts and decreases the complications associated with CVC insertion, especially for the IJV, and should become the standard of care in intensive care.

## Introduction

The insertion of central venous catheters (CVCs) has become an integral part of management of a critically ill patient. Access to the central circulation may be required for the administration of hyperosmotic or vasoactive compounds, parenteral nutrition, rapid infusion of large volumes of fluid or for the continuous or intermittent monitoring of biochemical or physiological parameters. Central venous catheter insertion is also indicated when the insertion of a peripheral line is not possible. Traditionally, CVC insertions have been performed using the landmark technique. The internal jugular, subclavian and femoral veins are commonly used sites. Even with experienced operators, complication rates of up to 12.3% have been reported for CVC insertion using the conventional landmark technique.[1] Considering the number of CVCs being inserted every day, this can amount to a large number of complications. Efforts to minimize and prevent the occurrence of complications should be a routine component of quality improvement programs.

There is an increasing body of evidence supporting the use of USG for CVC placement. The Agency for Healthcare Research and Quality in USA[2] and the National Institute of Clinical Excellence (NICE), in the United Kingdom[3] have recommended the use of USG guidance for CVC placement as one of the practices to improve patient care. In spite of these recommendations, the use of USG guidance when inserting CVCs has not gained much acceptance and is still used only sparingly, even in Western countries.[4]

Advances in specialty care in India and the availability of portable ultrasonography (USG) units in hospitals have made the use of USG for bedside procedures, possible in many institutes.[5] Our aim was to study the impact of USG on success rates and mechanical complications of CVC insertion.

## Materials and Methods

### Study design and setting

This was a prospective, randomized study performed in the medical and surgical intensive care units (ICU) of a tertiary care hospital.

### Selection of participants

All patients admitted to the ICUs between April 2007 and September 2008 and requiring central venous access as part of their management were enrolled in the study. Indications for CVC insertion included difficult peripheral venous access, need for invasive hemodynamic monitoring and delivery of inotropic medications or antibiotics. Informed consent was obtained from the subject or relatives before enrollment. The exclusion criteria included age below 18 years and refusal to give consent for inclusion in the study.

Using a computer-generated randomization chart, the patients were randomized into two groups: in the first group, CVCs were inserted using the anatomical landmark technique (ALT group) and in the second group USG guidance was used for inserting CVCs (the USG group).

### Interventions

Operators were categorized into two groups according to experience. Operators with less than 6 years of experience were classified as ‘registrars’ and those with more than 6 years of experience in the field of anesthesia and critical care were classified as ‘consultants.’

The right internal jugular vein (IJV) was the first choice for cannulation. Other sites such as the left IJV, left or right subclavian (SCV) or femoral veins were cannulated only if the right IJV was not available for cannulation due to the presence of a previously inserted CVC or dialysis catheter.

**Figure 1:**
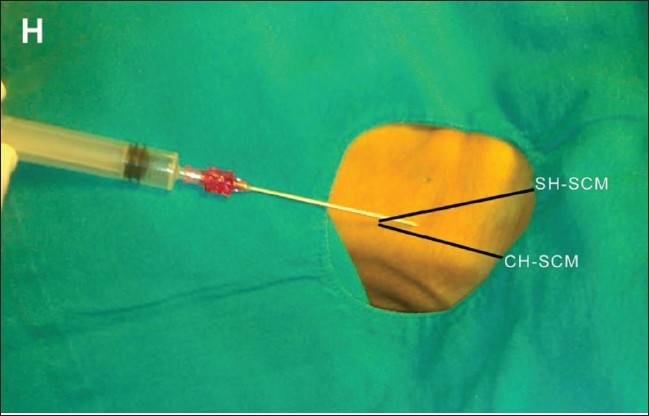
Insertion of a CVC in the right IJV using the anatomical landmark technique. The image shows the position of the needle with respect to the clavicular (CH-SCM) and sternal (SH-SCM) heads of the sternocleidomastoid muscle. H indicates the head end of the patient

**Figure 2:**
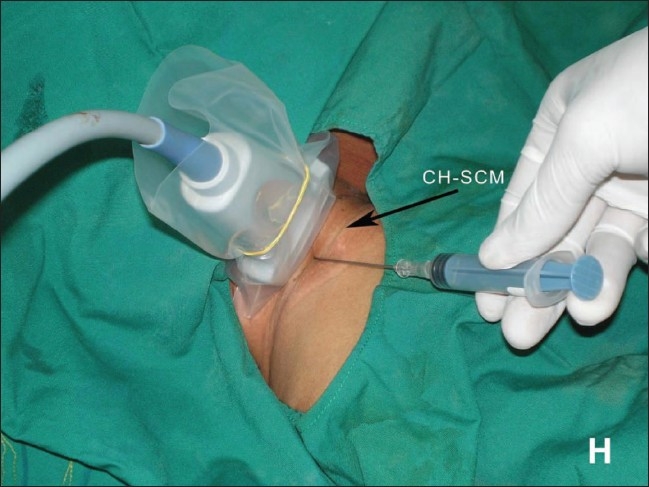
Insertion of a CVC in the right IJV under USG guidance. The image shows the position of the USG transducer and needle with respect to the clavicular head of the sternocleidomastoid muscle (CHSCM). H indicates the head end of the patient

**Figure 3:**
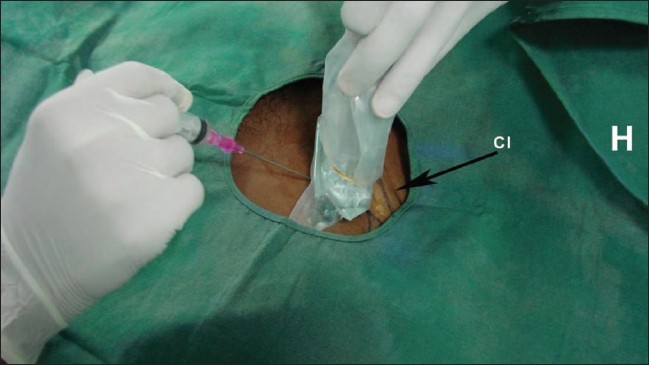
Insertion of a CVC in the right subclavian vein under USG guidance. The images shows the position of the USG transducer and needle with respect to the clavicle (Cl). H indicates the head end of the patient

**Figure 4:**
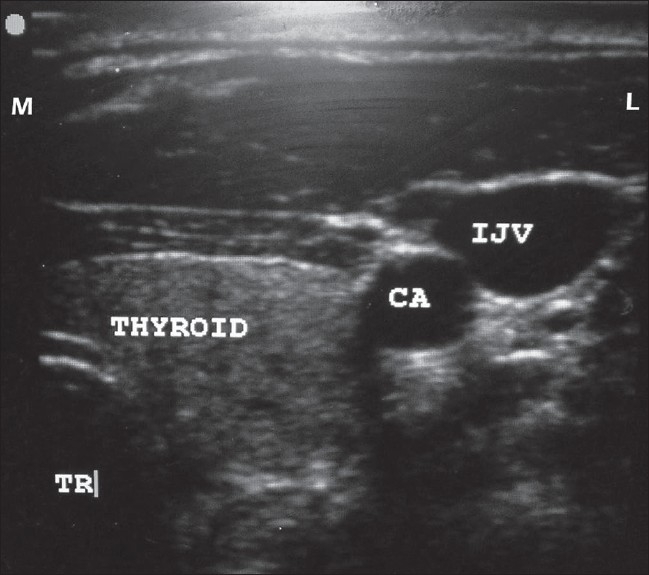
USG shows the location of the right IJV in relation to the carotid artery (CA), thyroid and trachea (TR). M indicates medial and L indicates lateral

Cannulation using the landmark technique was performed as per the standard guidelines [Figure 1].[6] Ultrasonography-guided catheters were inserted using a portable, software-controlled, USG system with a 13-6 MHz, 38-mm linear-array transducer (SonoSite Inc., Bothell, WA). To maintain sterility, the lead and transducer were cleaned with antiseptic solution and the probe was covered with a sterile sheath and gel. The patient was placed in the Trendelenburg position and the neck was sterilized with an antiseptic solution. For IJV cannulation, the transducer was placed perpendicular to the vessels at the apex of the triangle formed by the two heads of the sternocleidomastoid muscle and the clavicle [Figure 2]; for SCV cannulation, the transducer was placed slightly inferior to the middle third of the clavicle [Figure 3]. Although using transducer probe in the vertical plane has been recommended, we used it in the transverse plane as the vein and artery are best visualized in this plane and because the learning curve is less steep. The vein was identified by its large size and relation to the artery [Figure 4] and confirmed by checking its easy compressibility and by visualizing non-pulsatile, continuous flow using color Doppler. The transducer was positioned so that the vein was visualized in the center of the USG monitor.

The procedure was performed in real-time and hence a ‘seeker’ needle was not used; after giving local anesthesia, the introducer needle was directly inserted along the center of the probe towards the vein, under USG guidance. On the monitor, the needle could be seen either puncturing the vein or compressing the vessel. Once puncture of the vein occurred, we followed the modified Seldinger technique to insert a CVC. The position of the CVC was confirmed by a chest radiograph at the end of the procedure.

The procedure was considered a failure if the operator was unable to cannulate the vein within three attempts; an attempt being defined as the introducer needle's entry into the skin and its removal from the skin. If the initial method was unsuccessful after a maximum of three attempts, an alternative method was used, viz., USG was used if the insertion was being done by the ALT technique, help was taken from a more experienced operator or an alternative site was chosen.

At the end of the procedure, all data including patient demographics, reason for insertion of the CVC, level of operator experience, method of insertion, number of attempts and complications encountered were recorded.

### Outcome measures

The main outcome measure was the successful insertion of a CVC. Secondary outcome measures included the number of attempts and incidence of complications such as hematoma, pneumothorax, artery puncture, misplacement or nerve injury.

### Statistical analysis

We used SPSS, version 16.0 (SPSS Inc. Chicago, Illinois), for the statistical analysis. The means of continuous variables were compared using the Student's *t*-test and categorical variables were compared using the χ^2^ test and Fisher's exact test. We estimated the relative risks (RRs) for complications between the two procedures using the two sites of cannulation and also estimated the 95% confidence intervals (CI) around the point estimates and the reduction in risk. Additionally, we estimated the age-, sex- and operator-adjusted odds ratios (ORs) and the 95% confidence intervals around the point estimates for complications in the two groups. A *P*-value < 0.05 was considered statistically significant.

## Results

**Figure 5:**
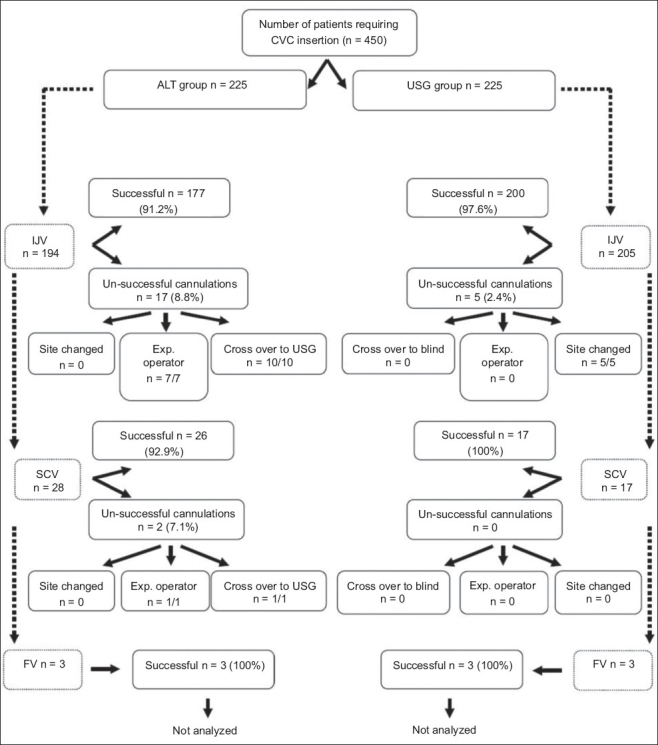
CONSORT diagram. CVC - central venous catheters, ALT - anatomical landmark technique, USG - ultrasound, IJV - internal jugular vein, SCV - subclavian vein, FV - femoral vein

A total of 450 patients were enrolled for the study, 225 each in the ALT and USG groups [Figure 5]. In the ALT group, 194 (86.2%) IJV, 28 (12.4%) SCV, and 3 (1.3%) femoral vein catheterizations were performed and in the USG group, 205 (91.1%) IJV, 17 (7.6%) SCV and 3 (1.3%) femoral vein catheterizations were performed. As the number of femoral vein catheterizations was small in both the groups, they were not included in the analysis. Patient characteristics are given in Table 1.

**Table 1:** Patient characteristics

Parameter of interest	Overall (*n* = 444)	IJV		*P* value (test)	SCV		*P* value (test)
					
		ALT (*n* = 194)	USG (*n* = 205)		ALT (*n* = 28)	USG (*n* = 17)	
Age (years ± SD)	48.9 ± 14	48 ± 13.9	49.3 ± 14.1	0.41 (t-test)	50.9 ± 13.9	51.1 ± 12.6	0.96 (t-test)
Sex, number of males (%)	299 (67.3%)	117 (60.3)	156 (76.1)	0.001* (χ^2^)	13 (46.4%)	13 (76.5)	0.048* (χ^2^)
Side cannulated, right side cannulation (%)	399 (89.9%)	178 (91.8%)	182 (88.8%)	0.318 (χ^2^)	23 (82.1%)	16 (94.1%)	0.25 (χ^2^)

* Statistically significant. IJV - internal jugular vein, SCV - subclavian vein, ALT - anatomical landmark technique, USG - ultrasound.

The main outcome measures for each group are summarized in Table 2. With regard to IJV cannulations, 177/194 (91.2%) were successfully inserted by the landmark technique and 200/205 (97.6%) were successfully inserted under real-time USG guidance (χ^2^ *P* = 0.006). The relative risk (RR) for unsuccessful IJV cannulation was 1.75 (95% CI: 1.18–2.59) for the ALT group as compared to the USG group; the risk reduction in the USG group being 11.71% (Fisher's exact *P* value = 0.005). On the other hand, for SCV cannulations, there were 26/28 (92.9%) successful insertions by the landmark technique and 17/17 (100%) successful insertions under USG guidance (χ^2^ *P* = 0.52). The RR for unsuccessful SCV cannulation was 1.62 (95% CI: 0.50–5.28) for the ALT group as compared to the USG group; the risk reduction in the USG group being 10.92% (Fisher's exact *P* value = 0.49). There was no significant difference between the failure rates based on operator experience in the ALT and USG groups for both IJV (*P* = 0.43) and SCV (*P* = 0.47) cannulations [Table 3].

**Table 2:** Outcome measures for CVC insertion

Outcome measure	IJV (*n* = 399)			SCV (*n* = 45)		
		
	ALT *n* = 194	USG *n* = 205	Difference (USG - ALT)	ALT *n* = 28	USG *n* = 17	Difference (USG – ALT)
Successful insertion	177 (91.2%)	200 (97.6%)	6.4%	26 (92.9%)	17 (100%)	7.1%
Success on first attempt	141 (72.7%)	173 (84.4%)	11.7%	20 (71.4%)	14 (82.4%)	11%
Average no. of attempts	1.5	1.2	−0.3	1.5	1.2	−0.3
Complication rates	19 (9.8%)	10 (4.9%)	−4.9%	4 (14.3%)	2 (11.8%)	2.5%

IJV - internal jugular vein, SCV - subclavian vein, ALT - anatomical landmark technique, USG - ultrasound, FE test - Fisher's exact test

**Table 3:** Comparison of operator experience with success rates

Parameter of interest	IJV (*n* = 399)				SCV (*n* = 45)			
		
	ALT (*n* = 194)		USG (*n* = 205)		ALT (*n* = 28)		USG (*n* = 17)	
				
	Inexperienced	Experienced	Inexperienced	Experienced	Inexperienced	Experienced	Inexperienced	Experienced
Lines inserted	136 (70.1%)	58 (29.9%)	164 (80%)	41 (20%)	21 (75%)	7 (25%)	15 (88.2%)	2 (11.8%)
Success rates	125 (91.9%)	52 (89.7%)	160 (97.6%)	40 (97.6%)	19 (90.5%)	7 (100%)	15 (100%)	2 (100%)
95% CI	87.3 to 96.6	81.6 to 97.7	95.2 to 99.9	92.6 to 100	76.8 to 100	NA*	NA*	NA*

* As the success rate is 100%, estimation of confidence interval is not applicable. IJV - internal jugular vein, SCV - subclavian vein, ALT - anatomical landmark technique, USG - ultrasound, CI - confidence interval.

The average number of attempts required for successful IJV catheter insertion was 1.5 (95% CI: 1.3–1.6) in the ALT group and 1.2 (95% CI: 1.1–1.3) in the USG group (*P* < 0.001). The average number of attempts required for successful SCV catheter insertion was 1.46 (95% CI: 1.1–1.8) in the ALT group and 1.18 (95% CI: 1–1.4) in the USG group (*P* = 0.23).

Internal jugular vein cannulation was successful in the first attempt in 173 (84.4%) patients in the USG group as compared to 141 (72.7%) patients in the ALT group (*P* = 0.004). For SCV cannulations, the first attempt was successful in 14/17 (82.4%) patients in the USG group as compared to 20/28 (71.4%) patients in the ALT group (*P* = 0.493).

The overall complication rate was 28/399 (7%) for IJV cannulation and 6/45 (13.3%) for SCV cannulation (χ^2^ *P* = 0.358). For IJV cannulation, complications were seen in 19/194 (9.8%) of patients in the ALT group and in 9/205 (4.4%) patients in the USG group (RR 2.22; 95% CI: 1.03–4.77); there was a risk reduction of 5.4%, with fewer complications under USG guidance (Fisher's exact *P* value = 0.04). The age-, sex- and operator-adjusted OR for complications was 0.35 (*P* = 0.03; 95% CI: 0.13-0.96) for the USG group, compared to the ALT group. In the ALT group, there were 9 (4.6%) carotid artery punctures, 10 (5.2%) hematomas and no (0%) pneumothoraces. In the USG group, there were 4 (2%) carotid artery punctures, 5 (2.4%) hematomas and no (0%) pneumothoraces.

For SCV catheterization, the complication rate was 4/28 (14.3%) for the ALT group and 2/17 (11.8%) for the USG group (RR 1.17; 95% CI: 0.24–5.74); the risk reduction was 2.5% (Fisher's exact *P* > 0.999). There was 1 (3.6%) arterial puncture, 1 (3.6%) hematoma, 2 (7.2%) mal-positioning and no (0%) pneumothoraces in the ALT group. In comparison, in the USG group there were no arterial punctures or hematomas but there was 1 case (5.9%) each of mal-positioning and pneumothorax. The age-, sex- and operator-adjusted OR for complications was 0.9 (95% CI: 0.16–5.0; *P* = 1.00) for the USG group compared to the ALT group.

Complication rates were significantly associated with the number of attempts. In the case of IJV cannulation, compared to a single attempt, the OR for complication with two attempts was 8.4 (95% CI: 3–23.6; *P* < 0.001) and for three or more attempts, the OR was 35.6 (95% CI: 12.5–101.6; *P* < 0.001). When two or more attempts were made for SCV cannulation, the OR for complications was 19.8 (95% CI: 1.7–221.6; *P* = 0.02).

There were a total of 22 unsuccessful IJV catheterizations with the initial method: 17 in the ALT group and 5 in the USG group [Figure 1]. These unsuccessful catheterizations were predominantly due to the inability to locate the vein in the ALT group and the inability to pass the guidewire in the USG group. Of the 17 unsuccessful cannulations in the ALT group, 10/10 (100%) were successfully performed using USG and 7/7 (100%) were successfully performed on the same side by a more experienced operator. On the other hand, for all five unsuccessful cannulations in the USG group, the cannulations had to be performed at some other site under USG guidance as either the vein was poorly visualized or the guidewire could not be passed. For SCV catheterizations, there were only two unsuccessful catheterizations in the ALT group; one of these was later successfully performed by an experienced operator and the other was performed successfully under USG guidance.

## Discussion

Central venous access has become an integral part of management of critically ill patients. These patients may require vascular access for either administration of fluids and drugs or for diagnostic hemodynamic monitoring. Central venous catheter cannulation is associated with a number of technical complications. The common ones are arterial puncture (10.6–13%),[7,8] hematoma formation (4–8.4%),[7,8] brachial plexus injury (1.7%),[9] pneumothorax (0–6.6%)[10,11] and hemothorax (1%).[12] The procedure is also associated with some rare but serious complications, including arterial rupture (<1%),[13] arteriovenous fistula formation (0.2%),[14] guidewire loss (0.5%),[15] chylothorax and chylopericardium.

With increased availability of portable USG units, USG-guided intervention is fast gaining acceptance as a valuable tool in the critical-care setting. Ultrasonography-guided procedures can save time and increase the accuracy, safety and efficacy of many interventions commonly performed in ICUs, including CVC insertion. There is abundant evidence that USG-guided catheter placement increases the safety and efficiency of the procedure. The benefits of using USG guidance over ALT for CVC insertion have been reported as far back as 1978,[16] and the body of literature supporting the use of USG continues to increase. The advantages of USG guidance over ALT in CVC insertion include risk reduction,[2,8,9] improved success rates,[2,9,17] quicker insertion,[8] a reduction in the number of attempts required[8] and the ability to cannulate in difficult situations.[18] The use of USG can ensure higher success rates in children and infants,[17,19] even when cannulation is done by less experienced operators.[20,21] The use of USG can also obviate the need for a postprocedure chest radiograph and thus reduce costs and help avoid radiation exposure.[22] Since the risk of catheter-related blood-stream infections (CRBSI) rises with an increase in the number of attempts, the rates of CRBSI may be lower when CVCs are inserted under USG guidance.[8]

### Internal jugular vein catheterization

Insertion of IJV catheters using the landmark technique may be associated with high failure rates of up to 35%.[23] On the other hand, for USG-guided IJV lines, success rates ranging from 81.3 to 100% have been reported.[24,25] The image quality offered by 2-dimensional USG allows the user to clearly see variations in anatomy and to assess the patency of a target vein. Ultrasonography also shows the position of the needle relative to the vein and its surrounding structures, which can result in a lower technical failure rate, reduction in complications and faster access. Up to a 35% reduction in failure rates has been reported with the use of USG for the insertion of IJV lines.[23] Our study had a success rate of 91.2% for IJV cannulation by the landmark technique, which is consistent with other reports;[8,9,26] the success rate was higher (97.6%) for cannulation under USG guidance.

Intensive care medicine is an applied specialty and, sooner or later, technologies developed in other fields of medicine make their way into this field and come into routine use; USG is no exception. In the modern healthcare setup, radiological expertise is a precious commodity. With practice and training, nonradiologists can learn to use USG for such procedures as central line placement, leaving the radiology specialist free for other vital diagnostic and therapeutic duties. In this study, CVC cannulations with USG guidance were performed by anesthetists/intensivists. Although other series' have found that success rates can vary with the experience of the operator, especially for the landmark technique,[18,27] we did not find any such difference. This discrepancy may be explained by the difference in definitions; in the other studies, operators were classified as experienced if they had performed more than 25 cannulations, whereas we categorized operators on the basis of years of experience: i.e., ‘registrars’ with 0–6 years experience and ‘consultants’ with more than 6 years of experience in the field of anesthesia and critical care. Many of those in the registrar group had adequate experience and skills in performing CVC catheterizations and, hence, the success rates were comparable in the two groups.

There was a low incidence of mechanical complications for IJV cannulations using the USG-guided technique in our study (4.4%), which is in agreement with previous reports.[8,9,28] Also, as compared to other series', our study showed higher rates of successful cannulation in the first attempt and a lower average number of attempts required for successful cannulation under USG guidance.[8,9,18,23,27]

The risk for complications increases with the number of insertion attempts. McGee and Gould[29] reviewed the complications of CVC and stated that the incidence of mechanical complications after three or more insertion attempts was six times as compared to an insertion in the first attempt. In the present study, compared to a single attempt, the OR for complication after two attempts was 8.4 (95% CI: 3–23.6; *P* < 0.001) and it was 35.6 (95% CI: 12.5–101.6; *P* < 0.001) when there were three or more attempts at IJV cannulation.

### Subclavian vein catheterizations

Unlike for IJV cannulations, the evidence in support of USG guidance for SCV cannulations is limited.[30] A large randomized trial comparing USG guidance with the landmark technique found that the former had no effect on the rate of complications or failures; however, real-time USG guidance was not used for placement of the catheter in that study. The authors reported a complication rate of 9.7% in the USG group and of 9.8% in the ALT group. There were 12.4 and 12% failed attempts in the USG and ALT groups, respectively.[31] In our study, the success rates were 92.9 and 100%, and the complication rates were 14.3 and 11.8%, for cannulations in the ALT and USG groups, respectively. Lefrant *et al.*[32] reported a similar success rate of 90.5% and a complication rate of 15.1% in a large population undergoing SCV cannulation by the landmark technique.

The number of needle passes has been strongly associated with the rates of failure and complications. Mansfield *et al*.[31] found that the complication rate rose from 4.3% with one pass to 24% with more than two passes. In our study, the odds for complications were 19.8 times greater when there were more than two attempts.

We did not find any statistically significant difference in success rates, complication rates, number of attempts or number of successful first attempts between the USG and ALT groups. This may be due to lack of sufficient power in our study because of the small sample size. Although other studies have also reported similar results, the sample sizes in these studies too have been small, with around 50 subjects in each;[21,24,33] a larger trial is required to understand the role of USG in SCV catheterization.

In all 11 patients (10 receiving IJV and one SCV cannulation) in whom the initial ALT failed, the catheter was successfully inserted on the same side with the use of USG, indicating that placement of a catheter under USG guidance is technically easier as the procedure is performed under direct vision.

### Limitations

There were a few limitations to our study. Although an experienced operator was arbitrarily defined as one having more than 6 years of experience, this may not truly reflect the experience of the operator. Also, the number of patients having SCV catheters was too small for any conclusions to have much statistical significance. As the aim of the study was only to examine the technical complications related to CVC insertion, the impact of USG on CVC-related infections was not studied. The time required for cannulation was not compared between the two groups as there is some controversy over whether the time required for setting up of the USG machine and sterilizing the transducer is to be included as part of the procedure time; however, it seems logical to assume that with increasing experience the time required to cannulate under USG guidance will come down.

## Conclusions

Real-time USG-guided intervention is becoming an increasingly popular and valuable tool in the critical-care setting, where it can increase the accuracy, safety and efficacy of CVC insertion. Ultrasonography guidance reduces failure rates, is effective in eliminating multiple access attempts and reduces the risk of complications with IJV cannulation. A larger trial is required to study the role of USG in SCV catheterization. We recommend the use of USG to guide CVC insertion, especially in the IJV, and believe that it should become the standard of care in intensive care.
